# The effect of catheter rotation during its withdrawal on frozen thawed embryo-transfer cycles outcomes: A Case-control study

**DOI:** 10.18502/ijrm.v17i7.4859

**Published:** 2019-07-31

**Authors:** Maryam Eftekhar, Lida Saeed, Masrooreh Hoseini

**Affiliations:** ^1^Yazd Reasearch and Clinical Center for Infertility, Yazd Reproductive Science Institute, Shahid Sadoughi University, Yazd, Iran.; ^2^Afzalipour Hospital, Kerman University of Medical Sciences, Kerman, Iran.

**Keywords:** Embryo transfer, Assisted reproductive technique, Implantation, Pregnancy.

## Abstract

**Background:**

Embryo transfer (ET) is the last and the most clinical process in assisted reproductive technology cycle. It has been suggested that cervical mucus interacts with an adequate embryo transfer in different ways. A few studies showed that catheter rotation could discharge mucus entrapped in the embryo to neutralize embryo displacement.

**Objective:**

The aim of this present study was to compare the outcome of frozen embryo transfer (FET) based on catheter rotation during withdrawal.

**Materials and Methods:**

In this case-control study, the clinical documents of 240 women who experienced frozen embryo transfer cycles were reviewd. The subjects were divided into two groups (n = 120/each), including A) the rotation treatment group (360°) that underwent ET using catheter rotation and B) the control group including the subjects who experienced ET with no catheter rotation. Clinical and chemical pregnancies and implantation rates were compared between two groups.

**Results:**

Results showed that there is no significant difference between the basic clinical and demographic features of both groups (p > 0.05). A significant difference was observed in terms of the rate of chemical pregnancy between groups (21.7% vs 43.3%, p = 0.001 respectively). In addition, the rate of clinical pregnancy was significantly higher in study group than the control (33.35% vs 14.2%, p = 0.002 respectively).

**Conclusion:**

Our results demonstrated that catheter rotation during withdrawal increased the implantation rate and clinical pregnancy.

## 1. Introduction

Satisfactory implantation needs an effective embryo transfer (ET) technique, a suitable endometrium, and a high quality embryo (1). In assisted reproductive technology (ART), ET is considered as the ultimate and the most clinical process (1). The factors crucial in affecting the success of in vitro fertilization (IVF)-ET programs include the important dimensions of embryo replacement, such as the existence of mucus or blood on the transfer catheter, dummy ET, catheter choice, guided transfer, ultrasonography, uterine contraction, practitioners' differences of approaches, as well as the ET problems (2).

Embryo replacement could be impaired seriously by cervical mucus (1). The presence of cervical mucus at the catheter's tip can prohibit embryos from exiting the catheter (3). In addition, it may pull the embryo off when removing the catheter. In case mucus is impelled or inserted in the uterine cavity, it could also interrupt implantation. In a trial dummy ET making use of Methylene Blue, it was shown that the dye got expelled at the external os at a remarkably greater rate in conditions where cervical mucus did not get withdrawn (4).

Mucins is the major elements of cervical mucus (3). The viscoelastic features of the body fluids, such as mucus, are normally evaluated using the rotational movement so as to calculate a shear stress value. Hence, it seems that the rotational movement is an intervention effective in creating shears upon mucins (5, 6). A few studies implied that catheter rotation could discharge mucus entrapped in the embryo so as to thwart embryo displacement (3).

Hence, in the present study, the frozen embryo transfer (FET) outcomes were compared on the basis of catheter rotation when withdrawn.

## 2. Materials and Methods

In this present case-control study, the clinical documents of 240 women who experienced FET cycles from April to October 2018 were examined. The subjects included had received intracytoplasmic sperm injections (ICSI)/IVF and experienced cryopreservation. woman aged over 42, the ones with body mass index > 30 kg/m${}^{2}$ as well as the individuals with the record of endocrine abnormalities and serious endometriosis were excluded from the research. Woman were divided into two groups (n = 120/each), A) the rotation treatment group (360°) that underwent ET using catheter rotation and B) the control group including the subjects who experienced ET with no catheter rotation. In the end, clinical and chemical pregnancies as well as the implantation rates were compared between two groups.

### Endometrium preparation

The endometrial preparation process was alike in the two groups.Woman were administered with oral estradiol valerate 6 mg/day (2 mg, Aburaihan Co., Tehran, Iran) from the 2${}^{\mathrm{nd}}$ day of menstrual period. Vaginal ultrasonography was utilized to measure endometrial thickness. Since the 13${}^{\mathrm{th}}$ day of period, when the thickness of the endometrium reached 8 mm, each patient was administered with CyclogestⓇ vaginal suppository (Cox Pharmaceuticals, Barnstaple, UK), 400 mg/twice a day, until the menstrual period or in eight weeks following the ET process in the event of clinical pregnancy. ET was carried out three days after the administering progesterone starts. Progesterone and estradiol were maintained up until 10${}^{\mathrm{th}}$ wk of the gestational age.

### ET procedure

All individuals were poisoned in a lithotomy state with a bivalve speculum entered inside the vagina, and the cervix was exposed. Using a saline solution, the cervix was cleaned. In addition, mucus was withdrawn using a suction pipet (Rampipella, RI.MOS. Mirandola, Italy). In the meantime, in the embryo culture lab, embryologists assessed the embryos thawed and placed or one or two of them in a soft catheter (Labotect GmbH, Rosdorf, Germany) with a fixed volume (30 µL) of a culture medium. The catheter was delivered by embryologists to a gynecologist carrying out the ET task. When the catheter was positioned in the suitable place, the embryos got mildly freed inside the uterine, and in the study group, the catheter got removed gradually with 360° rotation, yet without any rotation in the control one. The embryologists checked the catheter forthwith by a microscope so as to assure that the embryos were not retained. The probable traces of mucus or blood in the cannula was recorded. The transfer was regarded easy if the catheter entered the uterine with no cervical manipulation, making no use of forceps. Whereas, the transfer was regarded difficult in case it entered the uterine making use of forceps.

### Pregnancy outcomes

Chemical pregnancy defined as serum β HCG > 50 IU/L, on day 14 following the ET. In the same vein, clinical pregnancy was confirmed by identifying fetal heartbeats 14 days following the positive β HCG. In addition, the gestational sac number in 100 transferred embryos defined the implantation rate.

### Ethical consideration

The ethics committee of the institute approved this study under the reference code IR.SSU.RSI.REC.1397.16. Because this study was retrospective we didn't have specific written consent and we said them oral

### Statistical analysis

Making use of the SPSS software package (Statistical Package for the Social Sciences, version 20.0, Chicago, IL, USA), the statistical analysis was performed. To identify meaningful differences between the two groups, both chi-square test and *t*-test were utilized. The significance level was determined at p < 0.05.

## 3. Results

The data on 240 qualified subjects were analyzed. The basic clinical and demographic features of both groups had no differences of significance (Table I). Likewise, the cases of blastocyst- and cleavage-phase ETs were of the same number in the two groups (p = 0.35). A significant difference was observed between both the groups in terms of the rate of chemical pregnancy (21.7% vs 43.3%, p = 0.001). In addition, in the study group, the rate of clinical pregnancy was significantly higher (33.3%5 vs 14.2%, p = 0.002). Blood and mucus were identified similarly on the catheter's tip (p = 1.00) (Table II). Only three and two retained embryos existed in the control group and in the case group, respectively.

**Table 1:** Cycle characteristics in frozen embryo transfer


**Variable**	**Rotation group**	**Control group**	**P-value**
Endometrial thickness (mm) **	9.19 ± 1.46	8.97 ± 1.37	0.24
No. of transferred embryos **	1.92 ± 0.39	1.90 ± 0.45	0.65
No. of difficult transfer*	5	5.8	0.78
Embryos grade*			
A	20	21.7	
B	65	67.5	
C	15	10.8	<brow>-3</erow> 0.62
Cleavage stage of embryos*			
Cleaved	80	75	
Blastocyst	20	25	<brow>-2</erow> 0.35
Mucus detection at tip of catheter	0.8	0.8	1
Blood detection at tip of catheter	6.7	6.7	1
*Data presented as percentages. Student *t*-test **Data presented as mean ± SD. Chi-square			

**Table 2:** ART outcome


**Variable**	**Rotation group**	**Control group**	**P-value**
Implantation rate*	18.47	8.19	0.002
Chemical pregnancy rate/transfer**	52 (43.3)	26 (21.7)	0.001
Clinical pregnancy rate/transfer**	40 (33.3)	17 (14.2)	0.002
*Data presented as percentages **Data presented as n (%)			
Chi-square			

## 4. Discussion

The hypothesis of this study implies that rotating the catheter when it is being withdrawn may lead to an increase in the implantation rate, though inhibiting mucus remained from getting entrapped and leading to mucus-associated embryo displacement. Even if the results demonstrated no difference between the two groups concerning blood and mucus at the catheter's tip, the results implied remarkably higher clinical and chemical pregnancy rates in the group with catheter rotation.

The traces of mucus at the catheter's tip is supposed to lead to descending embryo displacement from the transferred surface in the process of withdrawing the catheter (7). In the clinic for this study, we regularly cleaned cervical mucus prior to the insertion of the catheter using the suction pipet, so only one case was reported that had mucus detected in the catheter in every group. However, the reason could be that only a small trace of mucus might lead to embryo's displacement.

The goal must be the placing of embryos in the uterus so accurately and meticulously as to get prepared for suitable fetal development and implantation. To ensure that all the steps in the process of embryo transfer is taken properly, separate elements should be assessed individually. Because of the contradictory findings in the medical literature, the impact of withdrawing cervical mucus before ET is a highly controversial debate. Some studies have reported enhanced rates of pregnancy (8-10), while others have reported no enhancement (11, 12). The other advantage of mucus removal and cervical cleaning is the reduction of the bacterial contamination risk in the endometrial cavity and the catheter (8). It has been shown that when the catheter's tip is contaminated with microorganisms, including streptococci (groups D and B), *E. coli*, mycoplasma, ureaplasma, and staphylococci (13), even when prophylactic antibiotics are used, pregnancy and implantation rates are reduced. Therefore, the cleaning of the cervical canal could exert a positive effect through lowering the risk of pathogens' entering the endometrium and changing the cervical environment (8).

The research by Abide and colleagues in the literature review, was found to be similar to the present one. In a randomized prospective trial, they juxtaposed the ET on the basis of the rotation of the catheter when withdrawn in the cases of unexplained infertility. Two hundred ICSI patients experiencing ET with blastocyst- or cleavage-stage fresh embryos were divided randomly into two groups; the first group with (n = 100) and the second one without (n = 100) catheter rotation when withdrawn; the rate of pregnancy was significantly higher in the research group (41% vs. 26%, p = 0.04), and the rate of clinical pregnancy was remarkably higher in the research group (39% vs. 25%, OR 1.9 [1.1-3.5], p = 0.05). Nevertheless, the running rate of pregnancy was similar in the two groups (33% vs. 23%, p = 0.2) (3).

Eskandar and colleagues conducted a controlled prospective trial in order of determine the impact of the withdrawal of cervical mucus before the transfer of the embryo. They reported that the mild withdrawal of cervical mucus exerted a positive effect on the rates of clinical pregnancy and embryo implantation, despite the increase in the level of the difficult ET. They also asserted that remarkably higher rates of patients experiencing the removal of cervical mucus before ET underwent clinical pregnancy. In addition, they reported that cervical aspiration before ET could be conducted regularly in those who experience ET via the cervical path (8).

Aflatoonian and colleagues selected 340 subjects randomly without or with mucus aspiration. They reported that the aspiration of cervical mucus using insulin syringes prior to the ET could boost the rate of pregnancy (9).

A major concern with ET could be the probability of embryos getting expelled. Making use of a radiopaque dye in order of mimicking ET, Knutzen and colleagues identified that a contrast remained following ET, principally in the uterine in just 58% of the subjects. In addition, it was observed that the rest of the subjects lost the chance of pregnancy due to the embryo expulsion (14).

Since we removed mucus before embryo transfer in all cases, maybe another factor causes increased pregnancy rate in the rotation group. Rotation probably induces a force contrary to the capillary force that causes release embryos in suitable site. Embryos could also return inside the cervix because of the capillary action where the injected fluid trailed the catheter withdrawn. In this regard, Madani and co-worker (15) introduced an extra volume of air following the injection of the embryo fluid. This additional volume of air led to a remarkable improvement in the rates of pregnancy and implantation. It is worth noting, nevertheless, that the appearance of the bubbles of air would not fix the issue. Saravelos and colleagues (16) reported that 12.4% of 277 subjects experienced air bubbles having migrated in the direction of the cervix as evaluated by ultrasound 1 hr following the transfer; as a result, the rates of clinical implantation and pregnancy in these individuals were remarkably lower than in the ones with their embryo/bubbles having been fixed or migrated in the direction of the fundus.

## 5. Conclusion

The results of data analysis in this study demonstrated that the rotation of the catheter when withdrawn increased the rates of implantation and clinical pregnancy. More studies need to be conducted to verify the results of the current study, especially ongoing pregnancy and live birth rates.

##  Conflict of Interest

There is no conflict of interest in this study.
